# A lateral flow immunoassay test performance in SARS-CoV-2 seroprevalence surveys: a validation study among healthcare workers

**DOI:** 10.1080/22221751.2020.1852893

**Published:** 2020-12-01

**Authors:** Ronan Garlantézec, Christopher Heslan, Emilie Tadie, Pierre Tattevin, Vincent Thibault, Christophe Paris

**Affiliations:** a CHU de Rennes, Univ Rennes, Inserm, EHESP, Irset (Institut de recherche en santé, environnement et travail), Rennes, France; b CHU de Rennes, Rennes, France; c CHU de Rennes, Univ Rennes, INSERM U1230, Rennes, France

**Keywords:** SARS-CoV-2, COVID-19, serological test, immunoassays, rapid test

## Abstract

The objective of this study was to evaluate the validity and reliability of NG-Test® when used as a finger-prick test on healthcare workers and to compare it to the ELISA Wantai Immunoassay. Fifty-one healthcare workers who were RT-PCR SARS-CoV-2 positive and 59 who were RT-PCR SARS-CoV-2 negative accepted to participate in this study. They were subjected to an NG-Test® finger-prick test and collection of a blood sample on the same day. A second NG-Test® on another finger was performed for the first 30 cases and controls and read blinded to the first. Sera obtained from blood samples were used to perform the Wantai SARS-CoV-2 ELISA. The interobserver agreement for the NG-Test® test was perfect (kappa coefficient = 100% [98%–100%]). The sensitivity of NG-Test® was estimated to be 85% [71.9%–92.3%] and the specificity 98.3% [95.0%–100.0%]) for both IgG and IgM. The percentage of agreement between the Wantai immunoassay and NG-Test® was 92.73% for IgG (Kappa = 0.85 [0.75–0.95]) and 65.45% (Kappa = 0.42 [0.26–0.58]) for IgM. Our study highlights the need to validate rapid immunoassay tests under real-life conditions. If NG-Test® is used in seroprevalence surveys, we recommend that its diagnostic performance be taken into consideration to obtain a reliable estimation.

SARS-CoV-2 sero-epidemiological surveys are necessary to evaluate the rate of immunisation, to identify risk factors, and to adapt prevention strategies in the general population, as well as among workers exposed to, such as healthcare workers (HCWs). Such studies require valid, reproducible and easy-to-use immunoassay devices. In the December issue of Emerging Microbes and Infections, Dortet et al. [1] published a paper concerning the performance of NG-Test^®^ (NG Biotech Laboratoires, Guipry-Messac, France), a lateral flow immunoassay (LFIA) that allows the detection of both IgG and IgM against SARS-CoV-2. This validation was mainly made on sera from hospitalised patients with a documented COVID-19 infection, as documented by at least one positive SARS-CoV-2 RT–PCR, to study sensitivity and sera of negative patients and few healthy volunteers to study specificity. They found an excellent sensitivity (>95% at 15 days post symptom appearance) and specificity (100% (93.3%–100%)) for both IgG and IgM. In their discussion, Dortet et al reported that NG-Test^®^ can be used to monitor immunological status of HCW or the general population. We think however that it seemed appropriate to better assess its performance in a non-hospitalised population, who may present a different immune response than what seen in hospitalised patients, before conducting a large sero-epidemiological survey. The objective of the present study was therefore to evaluate the validity and reliability of the NG-Test^®^ immunoassay in an HCW population and to compare it to the Wantai Ab Immunoassay, a well validated ELISA immunoassay [2–4].

We conducted a validation study at the Rennes University Hospital, a 1500-bed tertiary care centre in western France. 51 HCWs who were SARS-CoV-2 RT–PCR positive (cases) agreed to participate in this study. In addition, 59 HCWs with a negative SARS-CoV-2 RT–PCR (tested in some specific wards when at least one patient or one HCW in these units was diagnosed with COVID-19), with no history of symptoms before inclusion and no known contact with COVID-19 cases agreed to participate. All cases and controls were included in the present study between May 29 and July 3, 2020. At inclusion, all participants signed an informed consent form. On the same day, they underwent both an NG-Test^®^ finger-prick test and collection of a venous blood sample (7 mL) in the occupational medicine unit. As recommended by the manufacturer, the NG-Test^®^ was read 20 min after the finger prick by a trained nurse. To evaluate interobserver agreement, a second nurse performed a second NG-Test^®^ on the first 30 cases and the first 30 controls on another finger and read it blinded to the results of the first test. Serum samples obtained from the venous draw were used to perform the Wantai SARS-CoV-2 Ab ELISA for all cases and controls. To address the influence of the starting material, 10 µL of serum from all cases with a previously documented positive RT–PCR that were negative by the NG-Test^®^ on capillary blood were tested with an additional NG-Test^®^ at the Virology laboratory. We studied interobserver agreement by estimating the Kappa coefficient with 95% confidence intervals. Sensitivity and specificity of the NG-Test^®^ and the Wantai ELISA immunoassays were estimated with their 95% confidence intervals for all cases and controls using the documented RT–PCR results as the gold standard for case classification. The percentage of agreement between both immunoassays was calculated as well as the corresponding Kappa coefficient estimates.

Cases and controls were mainly women (72% and 83% respectively), aged < 50 years (76% and 79% respectively) and working as nurses or nurses’ aides (64% and 68% respectively). Among cases, the median number of days between the SARS-CoV-2 RT–PCR positive results and participation in this study was 61 [min: 32 days – max: 94 days]. The most frequently observed symptoms among cases were hyposmia or hypogeusia (85%), fatigue (81%) and headache (80%). All HCWs reported at least one symptom and none were hospitalised for COVID-19.

The interobserver agreement of the NG-Test^®^ test was perfect: the second blinded finger-prick test among the 30 cases and 30 controls showed similar results to the first test, leading to a kappa coefficient of 100% [98%–100%]. For both IgG and IgM, the sensitivity of NG-Test^®^ was equal to 85% [71.9%–92.3%] whereas the specificity was equal to 98.3% [95.0%–100.0%] (Table 1). The Wantai Immunoassay sensitivity was equal to 96.1% [90.8%–100%] for IgG but 35.3% [22.2%–48.4%] for IgM whereas the specificity was equal to 100% [93.4%–100%] for both IgG and IgM. Among the seven cases positive by RT–PCR but negative for IgM and IgG by NG-Test^®^ on capillary blood, the second NG-Test^®^ performed on sera gave concordant results (all 7 negative for IgG and IgM). The Wantai immunoassay and NG-Test^®^ showed 92.73% concordance for IgG (Kappa = 0.85 [0.75–0.95]) and 65.45% concordance for IgM (Kappa = 0.42 [0.26–0.58]).

**Table 1.** Performance of NG-Test^®^ and the Wantai ELISA immunoasay for SARS-CoV-2 antibody detection in Healthcare Workers (HCWs), Rennes University Hospital, France 2020.

		IgG	IgM	IgG or IgM
**LFIA NG-Test**^®^**(vs RT-PCR)**				
	TP	42	42	42
	FN	9	9	9
	Sensitivity	82.5% [71.9%–92.3%]	82.5% [71.9%–92.3%]	82.5% [71.9%–92.3%]
	TN	58	58	58
	FP	1	1	1
	Specificity	98.3% [95.0%–100.0%]	98.3% [95.0%–100.0%]	98.3% [95.0%–100.0%]
**Elisa Wantai (vs RT-PCR)**				
	TP	49	18	49
	FN	2	33	2
	Sensitivity	96.1% [90.8%–100%]	35.3% [22.2%–48.4%]	96.1% [90.8%–100%]
	TN	59	59	59
	FP	0	0	0
	Specificity	100% [93.4%–100%]	100% [93.4%–100%]	100% [93.4%–100%]

TP: true positive, FN: false negative, TN: true negative, FP: false positive.

The sensitivity and specificity reported in our study for the NG-Test^®^ are lower than those reported in three recent publications [1,5,6], all conducted retrospectively on sera. One explanation for the lower sensitivity and specificity observed in our study relative to these three studies may be the different characteristics of the included participants: the vast majority of participants in our study were women <50 years of age, whereas the cases were mainly men over 50 in the other three studies (median age = 58 years for Dortet et al.) [1,5,6]. In addition, the clinical presentation of COVID-19 was also different in our population: no HCWs required admission for COVID-19, whereas most participants in the three published studies were hospitalised (up to 82% in the study of Dortet et al.) [1,5,6]. Several publications have suggested that the immune response may be stronger in older male patients with severe forms of COVID-19 [7–9]. Consequently, the enrolment of participants with exclusively non-severe COVID-19 in our study may explain the reduced ability of NG-Test^®^ to detect IgG or IgM in our population. Another explanation for the discrepancies between the studies may have been the matrices; serum remains the reference material for serological testing, whereas capillary blood by its nature contains less Ab. However, retrospective testing of sera of all negative NG-Test^®^ cases gave concordant results.

The sensitivity and specificity of the Wantai Elisa immunoassay in our study were in the same range as those obtained in previous studies (i.e. sensitivity > 95% and specificity = 100%) [2–4] at least 15 days after symptom onset. The agreement with NG-Test^®^ was excellent for IgG but only moderate for IgM. Moreover, the rate of positive IgM (82.5%) detected with NG-Test^®^ in cases is quite surprising, given that IgM are reported to wane after seven weeks [7,10], whereas the median time since the SARS-CoV-2 positive RT–PCR was 61 days (more than 8 weeks) in our study. This suggests that the NG-Test^®^ results for IgM must be interpreted with caution. This result is in accordance with that of the study of Charpentier et al. [6], which reported lower specificity than the two other studies on sera.

Our study highlights the need to validate rapid LFIAs under real-life conditions on the populations on which they will be used before carrying out large-scale seroprevalence surveys. Although our study showed excellent reproducibility of NG-Test^®^, the validation was not optimal, especially for sensitivity. Consequently, if the NG-Test^®^ is used in seroprevalence surveys among HCWs, we recommend taking into account its diagnostic performance to obtain a reliable estimation of the prevalence, as recommended [11]. Moreover, at an individual level, given that the PPV of the test may be low in settings with low SARS-CoV2 seroprevalence, a confirmatory test using ELISA immuno-assays would be warranted for such subjects.
